# Novel Screening Tool Using Non-linguistic Voice Features Derived from Simple Phrases to Detect Mild Cognitive Impairment and Dementia

**DOI:** 10.14283/jarlife.2023.12

**Published:** 2023-08-23

**Authors:** D. Mizuguchi, T. Yamamoto, Y. Omiya, K. Endo, K. Tano, M. Oya, S. Takano

**Affiliations:** 1 PST Inc., Yokohama, Japan; 2 Takeyama Hospital, Yokohama, Japan; 3 Honjo Kodama Hospital, Honjo, Japan

**Keywords:** Mild cognitive impairment, cognitive disorders, diagnosis, vocal biomarker, machine learning

## Abstract

Appropriate intervention and care in detecting cognitive impairment early are essential to effectively prevent the progression of cognitive deterioration. Diagnostic voice analysis is a noninvasive and inexpensive screening method that could be useful for detecting cognitive deterioration at earlier stages such as mild cognitive impairment. We aimed to distinguish between patients with dementia or mild cognitive impairment and healthy controls by using purely acoustic features (i.e., nonlinguistic features) extracted from two simple phrases. Voice was analyzed on 195 recordings from 150 patients (age, 45–95 years). We applied a machine learning algorithm (LightGBM; Microsoft, Redmond, WA, USA) to test whether the healthy control, mild cognitive impairment, and dementia groups could be accurately classified, based on acoustic features. Our algorithm performed well: area under the curve was 0.81 and accuracy, 66.7% for the 3-class classification. Thus, our vocal biomarker is useful for automated assistance in diagnosing early cognitive deterioration.

## Introduction

Alzheimer’s disease and other dementias (AD/D) are the most common chronic neurodegenerative disease worldwide. Mild cognitive impairment (MCI) is the prodromal phase of cognitive decline, a condition that can be reverted with proper interventions detected with neuropsychological tests (1). For all of these cognitive issues, early detection is essential to ensure effective and timely treatment and slow the progression of cognitive deterioration. For example, a growing consensus is that pharmaceutical interventions may be most effective at the earliest stages of dementia before serious and irreversible neuropathological changes begin (2).

Various screening techniques have been developed for detecting cognitive decline. Cognitive function tests such as the mini-mental state examination (MMSE) (3) and Montreal Cognitive Assessment (4) are conventional methods widely used to screen for AD/D and MCI. In addition, fluid biomarkers collected from cerebrospinal fluid, blood, saliva, and tears (5), and brain imaging with magnetic resonance imaging (MRI) (6) and positron emission tomography (PET) (7) are utilized as reliable clinical examinations to detect pathological findings such as the accumulation of amyloid β, which is a causative agent of AD. However, these methods have several disadvantages such as their time-consuming nature, high inspection cost, invasiveness, and the need for dedicated equipment.

As a relatively new approach, diagnostic assistance with the analysis of patient’s voice to detect cognitive deterioration (i.e., vocal biomarkers) has been extensively studied over the last decade (8). This approach is non-invasive, does not require specific or expensive equipment, and can be efficiently conducted remotely. In addition, voice data collection and analysis is reasonable price-wise, compared to brain imaging or fluid tests. Many studies have successfully detected cognitive impairments using voice data as vocal biomarkers. Most of the studies focused on binary classification tasks (healthy control vs. MCI or vs. AD); the accuracy for predicting AD ranged from 80 to 97%, while MCI ranged from 73 and 86%. Few studies tried 3-class classification tasks (healthy control, MCI, and mild stage of AD) in a model, which achieved an accuracy of 61% (9).

Most of the voice biomarker studies extract the prosodic and/or temporal features from the voice recorded during cognitive tasks such as picture descriptions (using the “cookie theft” picture in most instances) (10–13), sentence-reading tasks (14–17), and telling stories or having a conversation with a clinician (18–21), all of which are slightly time-consuming and require a skilled examinator to impose a task. In addition, when a patient is examined by using the same task repetitively to monitor the patient’s cognitive function, the task-based recordings could be highly affected by the “learning effect”. Thus, repeated exposure to the same task could mask cognitive decline (e.g., an individual remembers the answers in a task) (22).

Another common method is a machine-learning model with linguistic features that primarily uses natural language processing (NLP) (9,11,12,23). Although these methods offer high performance in dementia detection, their linguistic features are highly language-dependent. Thus, text-based models can be applied to limited regions where patients use the same language as that used in the regions in which the model is trained.

In this study, we aimed to test the performance of prediction models for detecting cognitive dysfunction using purely acoustic features (i.e., without linguistic features). Our model uses prosodic and temporal features from two simple phrases, that could be applied to patients in different regions with various languages.

## Methods

### Ethics statements

This study was approved by the local Ethics Committee for Research on Human Subjects in Japan (approval numbers, #000005 and #000006).

### Study participants

The participants of this prospective, observational study comprised 150 patients who were aged ≥45 years (up to 95 years) at the time of examination at two hospitals in Japan. All study participants provided informed consent and the research procedures were designed in accordance with the ethical standards of the committee described above and with the Helsinki Declaration of 1975 (as revised in 2000). Patients with respiratory infections and patients who did not understand or complete the assessment process were excluded. The participants were requested to complete two or three cognitive assessments: the Japanese version of the Montreal Cognitive Assessment (MoCA-J) (4,24), the revised version of the Hasegawa’s Dementia Scale (HDS-R) (25), and/or the mini-mental state examination (MMSE) (3). Based on the scores of these assessments, the participants were classified into one of three cognitive groups: healthy control (HE), MCI, and AD/D. The detailed classification criteria are listed in Table 1.

**Table 1.** Statistics of the demographic information and cognitive scores in the three groups (HE: healthy control, MCI: mild cognitive impairment, AD/D: dementia, MoCA-J: the Japanese version of the Montreal Cognitive Assessment, HDS-R: the revised version of Hasegawa’s Dementia Scale, MMSE: the Mini-Mental State Examination). Note that there was no subject with both MoCA-J ≥ 26 and HDS-R ≤ 20 (or MMSE ≤ 23)

	Group		
	HE	MCI	AD/D
Inclusion criteria	MoCA-J ≥26	MoCA-J ≤25 HDS-R ≥21 (or MMSE ≥24)	MoCA-J ≤25 HDS-R ≤20 (or MMSE ≤23)
N (% female)	13 (69.2%)	77 (70.1%)	105 (51.4%)
Age, y (mean±SD)	78.2±5.2	81.3±6.5	82.3±7.2
MoCA-J score (mean±SD)	27.2±1.4	20.4±2.7	12.3±5.5
MMSE score (mean±SD)	−	24.5±0.6	14.0±5.2
HDS-R score (mean±SD)	28.2±1.4	25.7±2.6	14.0±5.2

HE: healthy control, MCI: mild cognitive impairment, AD/D: Alzheimer’s disease and other dementias, MoCA-J: Japanese version of the Montreal Cognitive Assessment, HDS-R: the revised version of Hasegawa’s Dementia Scale, MMSE: Mini-Mental State Examination, SD: standard deviation; Note: No participant had both MoCA-J score ≥26 and HDS-R score ≤20 (or MMSE score ≤23).

### Sound recording

Sound recordings were obtained by using a directional pin microphone (ME-52W; OLYMPUS, Tokyo, Japan) connected to a portable, linear pulse-code modulation recorder (TASCAM DR-100mkIII; TEAC Corporation, Tokyo, Japan) at a sampling rate of 96 kHz with a 24-bit resolution. The microphone was attached to the patient’s clothes at the chest level, approximately 15 cm from the mouth. The patients were asked to utter two simple phrases: 1) sustain the vowel sound (/a/) for more than three seconds and 2) repeat the trisyllable (/pa-ta-ka/) five times or more as quickly as possible. We chose these two phrases because they have been used for various clinical assessments (26) and because such language-independent phrases have great usefulness in prediction models to be applied in different countries. In some instances, the patient’s voice was recorded more than twice on different days (2–5 times, with an adequate interval between recordings), thereby resulting in 195 sound recordings from 150 participants.

### Feature extraction

After the audio signals were downsampled to 16 kHz with 16-bit resolution, 17 acoustic features were extracted, including the statistics of pitch(F0)-related or voice quality-related features (e.g., shimmer, jitter, and harmonics-to-noise ratio) derived from the sustained vowel (/a/) and peak intensity-related features derived from the waveform of the repeating trisyllable (/pa-ta-ka/).

For the calculation of pitch-related or voice quality-related features, the audio signal was processed for each 10 msec window length. For the intensity-related features, peaks in the waveforms were extracted by calculating the relative maxima in the time series data of intensity values.

### Machine learning

LightGBM (Microsoft, Redmond, WA, USA), a gradient-boosting tree algorithm for classification, was used to create the machine-learning models. The objective function of the LightGBM was set to “multiclass” to predict the three classes: HE, MCI, and AD/D. The sample size in the HE group was smaller than that in the other two groups; therefore, we applied the synthetic minority oversampling technique (SMOTE) (27) to balance the sample size between targets in the training dataset. The hyperparameters for the LightGBM classifiers were optimized using the Optuna hyperparameter optimization framework (Preferred Networks, Tokyo, Japan). The following optimized parameters were used to build and evaluate the models: “learning_rate”, 0.01; “lambda_l1”, 0.0188; “lambda_ l2”, 0.00361; “num_leaves”, 31; “feature_fraction”, 0.4; “bagging_fraction”, 1.0; “bagging_freq”, 0; “min_child_ samples”, 5.

For the model evaluation, we applied five-fold group cross-validation. The data were randomized and split into five folds, one of which was used iteratively as the test set. The rest were used as the training set. All data from a given participant were categorized in the test set or training set, but not in both, to eliminate potential bias owing to identity-confounding factors. The area under the receiver operating characteristic curve (AUC) was analyzed to evaluate model performance. The average of the three one-vs-rest (OvR) AUCs and the classification accuracy, based on the confusion matrix, were calculated to test the overall performance of the prediction model in discriminating between the three classes. For each recording, the prediction class (shown in the confusion matrix) exhibited the highest prediction probability.

### Statistical analysis

Statistical analyses were performed by using R (version 4.1.2; R Foundation for Statistical Computing, Vienna, Austria). Chi-squared test and one-way ANOVA were used to test the difference in sex ratio and age between the three classes, respectively. A p-value less than 0.05 after the Holm-Bonferroni adjustment was considered statistically significant.

## Results

**Figure 1.**
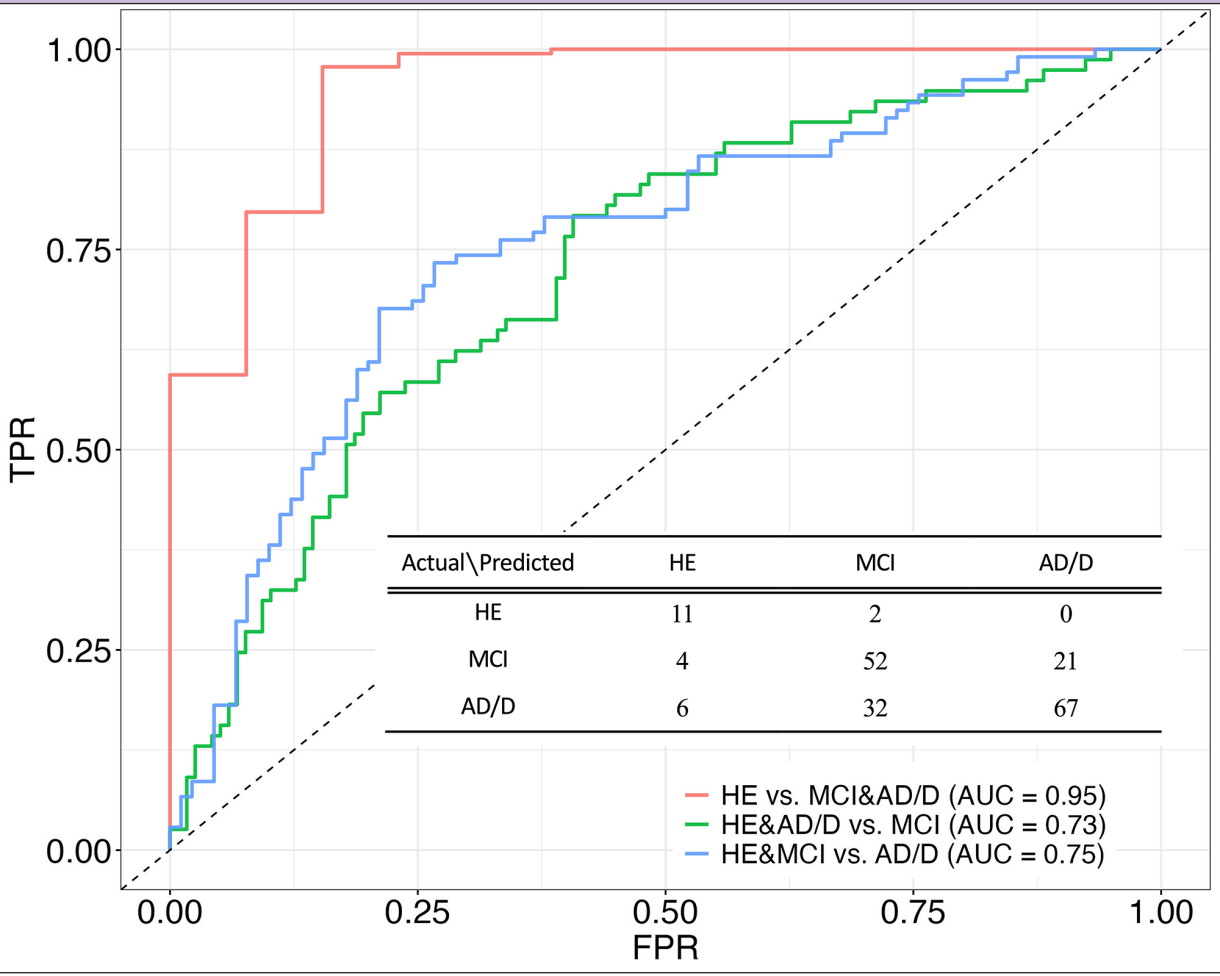
Three-class (one-vs-rest) receiver operating curves and confusion matrix derived from the machine learning model for the prediction of the three cognitive classes: healthy control (HE), mild cognitive impairment (MCI), and Alzheimer’s disease and other dementias (AD/D)

TPR: true positive rate; AUC: area under the curve, FPR: false positive rate

Figure 1 shows the three receiver operating characteristic curves derived from the three-class prediction model.

The average AUC (i.e., OvR discrimination) was 0.81. Among the three OvR AUCs, the highest was 0.95 when discriminating between HE and the other classes (i.e., MCI and AD/D). No significant differences existed between the three classes in sex ratio [chi-squared test, χ2 (1) = 2.46e-31, 0.84, and 5.69; p = 1, 0.72, and 0.051, for the HE vs MCI, HE vs AD/D, and MCI vs AD/D, respectively] or age [ANOVA, F(2) = 2.26, p = 0.11] (Table 1). The AD/D group predominantly consisted of patients with AD, followed by dementia with Lewy bodies and frontotemporal dementia.

All 17 acoustic features contributed to the prediction model. The LightGBM importance (gain) value ranged from 1021 to 2370 (AVE±SD = 1531±369), and the voice quality-related features (i.e., harmonics-to-noise ratio) showed the highest importance.

The accuracy score of the three-class prediction model was 66.7%, which was twice the chance level of the performance (33.3%). Given two-class prediction, predicting HE and the other classes (i.e., MCI and AD/D) achieved an accuracy of 93.8%, whereas predicting AD/D and the other classes (i.e., HE and MCI) achieved an accuracy of 69.7%.

## Discussion

In this study, we aimed to distinguish between patients with AD/D, MCI, and HE by using purely acoustic features, extracted from two simple phrases, and applying a machine-learning algorithm. We found that our algorithm performed well in distinguishing between the three groups. Increasing evidence indicates that pathological changes in dementia begin much earlier than the appearance of the clinical symptoms used to determine the onset of dementia (28). Speech alterations may be one of the earliest signs of such changes and are observed before other cognitive impairments become apparent (29). Previous studies have shown that voice quality-related features of speech (e.g., number of voice breaks, shimmers, jitter, and noise-to-harmonics ratio) reflect cognitive decline (15). Furthermore, changes in these features begin earlier during disease progression, and during the MCI stage. Our model also used such voice quality features and performed well in discriminating between the three classes (HE, MCI, and AD/D), which supports previous findings. Of note, although the sample size of the HE group was relatively small, our model showed the highest performance in discriminating healthy controls from the MCI and AD/D groups, given the binary classification. Thus, our model could be particularly useful for the early detection of cognitive decline during MCI.

To the best of our knowledge, this study imposed the most straightforward and simple task (utterance of two short phrases) to extract acoustic features and build a machine-learning model to predict cognitive impairments. Recording the two phrases (/a/ and / pa-ta-ka/) generally took less than 10 s, which is much shorter than cognitive tasks in previous studies (e.g., picture descriptions, sentence-reading tasks, and telling stories or having a conversation with a clinician). For the early detection of cognitive decline, monitoring cognitive changes frequently and continuously is essential, which is challenging in terms of adherence (30). Therefore, our simple task might contribute to maintaining the motivation of users to record their voices repeatedly, thereby leading to an assessment of trends in their cognitive function.

In conclusion, our findings demonstrate that purely acoustic features derived from two simple phrases have the potential to be one of the efficient tools for automatically assessing future dementia risk before other cognitive symptoms appear. Further research is required to test whether these acoustic features can discriminate between types of dementia (e.g., AD, dementia with Lewy bodies, and frontotemporal dementia) using larger and balanced datasets of audio samples. In addition, since our model was built with the data from only two hospitals in Japan, further validation should be conducted using sounds from patients whose first language is not Japanese, to test whether our model is not affected by the differences in the intonation or accents when uttering the two phrases (i.e., language-independent).

## Data Availability

The data are not publicly available due to personal information contained within.
